# Gut-bone axis crosstalk: Microbiota-driven immune-metabolic-neural networks in bone disorders and precision interventions

**DOI:** 10.1016/j.jot.2026.101126

**Published:** 2026-05-11

**Authors:** Rong Qin, Peng Yu, Hui Wang, Jing Zhou, Rui Gong, Yiyao Duan, Hongping Jia, Mingzhu Xie, Yucheng Zhou, Jun Hu

**Affiliations:** a Department of Gastroenterology, Yan’ an Hospital Affiliated to Kunming Medical University, Kunming, Yunnan, 650051, China; b Key Laboratory of Tumor Immunological Prevention and Treatment of Yunnan Province, Kunming, 650051, China; c Department of Orthopedics, Calmette Hospital Affiliated to Kunming Medical University, Kunming, Yunnan, 650034, China; d Department of Stomatology, Yan’ an Hospital Affiliated to Kunming Medical University, Kunming, Yunnan, 650051, China; e Department of Gynecology, Kunming Maternity and Child Care Hospital, Kunming, Yunnan, 650500, China; f Department of Comprehensive Surgical, Kunming Maternity and Child Care Hospital, Kunming, Yunnan, 650500, China

**Keywords:** Bone metabolism, Gut microbiota, Gut-bone axis, Osteoporosis, Probiotics, Short-chain fatty acids

## Abstract

The gut microbiota regulates bone metabolism via a complex gut-bone axis involving short-chain fatty acids (SCFAs), immune modulation, and neuroendocrine signals. However, the precise mechanisms remain unclear, and microbiota-targeted interventions (probiotics, prebiotics, fecal microbiota transplantation) are not yet optimized for clinical use. This review systematically synthesizes the immune-metabolic-neural interaction network within the gut-bone axis, highlighting non-linear crosstalk among SCFAs, bile acids, tryptophan derivatives, immune cells (macrophages, Treg/Th17), and vagus nerve signaling. We critically assess translational hurdles, including heterogeneous study designs, confounding factors, and lack of causal evidence. Based on this network perspective, we propose a framework for future research that prioritizes multi-omics approaches, stratified interventions, and rigorous trials. This synthesis advances understanding of how gut dysbiosis drives bone disorders and paves the way for precision skeletal medicine.

**Translational potential statement:**

This review identifies microbial markers for risk stratification of bone metabolic disorders and discusses SCFA-based strategies and fecal microbiota transplantation (FMT) in conditions including osteoporosis, impaired fracture healing, rheumatoid arthritis, and glucocorticoid-associated osteonecrosis. It provides testable hypotheses for large-scale randomized controlled trials (RCTs), directly supporting translation of microbiome research into clinical practice for bone disorders.

## Graphical abstract

**Figure:**
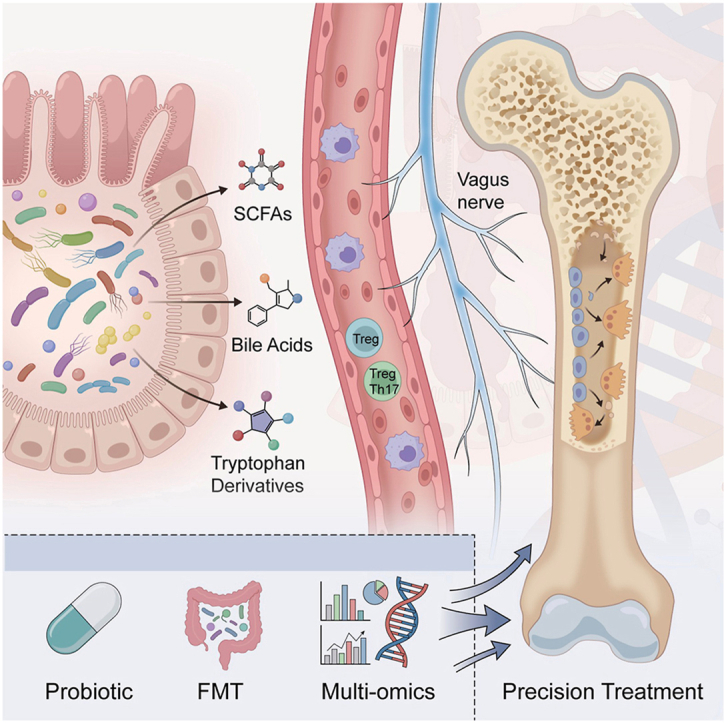

## 1 Introduction

The gut microbiota, often termed the ‘second genome,’ critically regulates skeletal homeostasis via the gut-bone axis [[1], [2], [3], [4]]. This axis orchestrates bone remodeling via a multifaceted network operating at metabolic, immune, and neuroendocrine levels. Metabolically, microbiota-derived SCFAs affect osteoclast and osteoblast functions in two ways: by inhibiting histone deacetylases (HDACs) and by activating G protein-coupled receptors (GPR43/41) [[5], [6], [7]]; the tryptophan metabolite melatonin (MLT) further enhances bone microarchitecture in osteoporosis models through intestinal barrier repair and modulation of the SCFAs/TMAO axis [[8], [9], [10]]. Immunologically, dysbiosis disrupts macrophage M1/M2 polarization and Treg/Th17 cell homeostasis, driving aberrant pro-inflammatory cytokine release (e.g., TNF-α, IL-17) that accelerates pathological bone resorption [11,12]. Neuroendocrinologically, gut metabolites (e.g., serotonin) integrate with vagus nerve signaling to form a “gut-brain-bone” circuit that finely tunes bone formation-resorption balance [13,14].

Disruption of this regulatory network directly underpins skeletal pathology. In postmenopausal osteoporosis (PMOP), estrogen deficiency induces gut dysbiosis, which exacerbates bone loss through gut barrier impairment and systemic inflammation [15,16]. Similarly, in high-fat diet (HFD)-induced obesity, diminished microbial diversity and increased intestinal permeability synergistically activate NF-κB signaling, promoting osteoclast hyperactivation [[17], [18], [19]]. This review outlines the various mechanisms involved in the gut-bone axis and critically evaluates translational challenges and prospects for targeted strategies, including probiotics and FMT, to advance precision management of osteoporosis and impaired fracture healing.

## 2 Fundamentals of gut microbiota and bone metabolism

### 2.1 Structural and functional organization of gut microbiota

The gut microbiota, which includes bacteria (mainly), fungi, viruses, and protists, creates a complex ecosystem essential for nutrient metabolism, immune balance, and hormone regulation [20,21]. These microbes directly modulate host physiology via bioactive metabolites including SCFAs, bile acids (BAs) and tryptophan derivatives (e.g., MLT) [22,23]: SCFAs (e.g., butyrate/propionate) suppress systemic inflammatory cascades by energizing intestinal epithelium and regulating macrophage polarization [22,[24], [25], [26]]; BAs mediate lipid digestion and signaling [27,28]; while tryptophan derivatives (e.g., indoles, melatonin) contribute to gut-bone signaling [2,29,30] Furthermore, dysbiosis (e.g., altered Firmicutes/Bacteroidetes (F/B) ratio, reduced diversity) correlates with osteoporosis (OP) progression [31,32], underscoring microbial homeostasis as critical for skeletal health. In summary, the influence of the gut microbiota on bone is not attributable to a single species or metabolite but is mediated synergistically by a network of metabolites (SCFAs, BAs, tryptophan derivatives). Consequently, the link between dysbiosis and osteoporosis should be conceptualized as a “network imbalance” rather than a mere shift in taxonomic abundance.

### 2.2 Physiological dynamics and regulatory control of bone metabolism

**Fig. 1:**
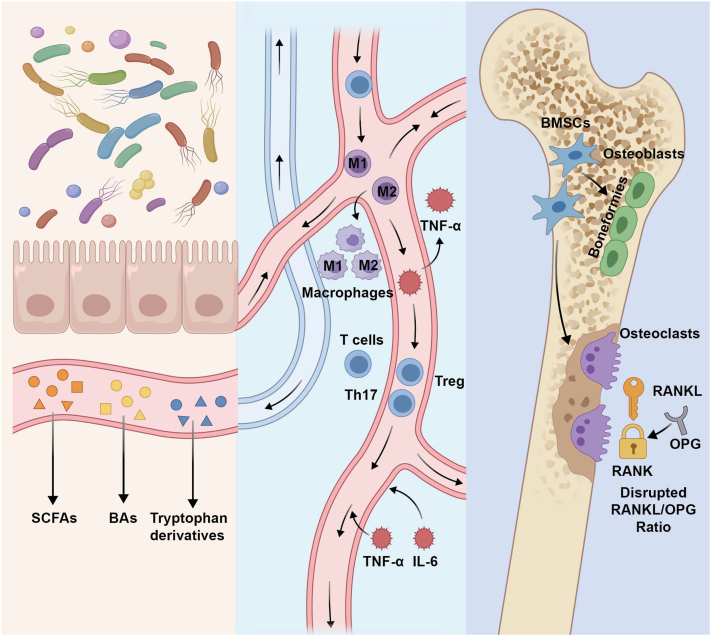
Schematic of the gut-bone axis.

Osteoblasts and osteoclasts dynamically regulate bone metabolism through a balance of formation and resorption [33], orchestrated through multi-tiered regulatory hierarchies [3,4]. Endocrinologically, estrogen deficiency elevates receptor activator of nuclear factor kappa-B ligand (RANKL)/osteoprotegerin (OPG) ratio to amplify osteoclast activity, precipitating postmenopausal bone loss [[34], [35], [36]]. Immunologically, pro-inflammatory cytokines (TNF-α, IL-6) accelerate resorption via NF-κB pathway activation [[37], [38], [39], [40]]. Microbiotically, the gut-bone axis regulates bone remodeling through SCFAs, immune modulation (e.g., Treg/Th17 balance), and gut barrier integrity, as detailed in Section 3 [3,[41], [42], [43]]. Therefore, Bone metabolism regulation is fundamentally a nonlinear interaction among the endocrine system, the immune system, and the microbiota. Focusing on any single component alone cannot adequately explain the whole process. An integrated “gut–bone axis” framework is therefore more appropriate for understanding the maintenance and disruption of bone homeostasis (Fig. 1).

## 3 Mechanisms of gut microbiota-mediated bone metabolism regulation

### 3.1 Direct effects of SCFAs on bone cells

SCFAs are the primary metabolites produced by microbial fermentation of dietary fiber [44,45]. Butyrate acts directly on bone cells: it inhibits HDACs, promotes Runx2/Osterix-mediated osteoblast differentiation and mineralization, and concurrently blocks nuclear factor of activated T cells c1 (NFATc1) nuclear translocation to inhibit osteoclastogenesis [[46], [47], [48]]. This direct action is independent of immune pathways and constitutes a major mechanism by which SCFAs regulate bone remodeling. Conversely, depletion of SCFAs caused by benzene exposure has led to bone metabolic disorders [[49], [50], [51]]. Beyond HDAC inhibition, the SCFA–GPR41/43 axis directly regulates lineage commitment of bone marrow mesenchymal stem cells (MSCs): acetate shifts MSC differentiation from osteogenesis toward adipogenesis via GPR41/43 activation, as reflected by downregulation of bone formation markers (for example, Col1a1) and upregulation of adipocyte markers (for example, Pparg), while Gpr41/43 double-knockout mice showed increased trabecular bone volume [52]. Thus, SCFAs exert dual effects: butyrate is osteoprotective, whereas acetate promotes adipogenesis via GPR41/43—a distinction crucial for precision targeting.

### 3.2 Immunoregulation and inflammatory response

The gut microbiota significantly influences bone metabolism by shaping the immune microenvironment, where the balance of macrophage M1/M2 polarization and the homeostasis of Treg/Th17 are central [53,54]. SCFAs indirectly modulate the immune system by activating GPR43/41, reducing TNF-α/IL-6 secretion in macrophages, and expanding the Treg population. This process inhibits bone resorption by restoring immune homeostasis [[55], [56], [57]]. MLT activates the Nrf2 pathway, which restores gut barrier integrity, lowers serum IL-17/TNF-α levels, promotes M2 macrophage polarization, and inhibits RANKL-mediated bone resorption [[58], [59], [60]]. In obesity-induced bone loss models, a HFD causes dysbiosis (↑F/B ratio), increasing gut permeability by disrupting tight junctions (↓ZO-1/Occludin). This allows lipopolysaccharide (LPS) to activate osteoclast precursor cell differentiation through the Toll-like receptor 4 (TLR4)/NF-κB pathway [61,62]. Fructooligosaccharides (FOS) and galactooligosaccharides (GOS) restore microbial α-diversity, increase butyric acid levels, strengthen epithelial tight junctions, and inhibit M1 polarization, thereby reversing bone loss [45,[62], [63], [64]]. Ultraviolet radiation enhances bone density by converting skin-derived vitamin D3 into its active form and inducing microbiota-dependent immunomodulation, such as increasing the Treg ratio [65]. These findings highlight a complex regulatory network within the microbiota-immune-bone axis, where SCFAs, MLT, and other microbial metabolites work together to regulate immune responses and maintain bone homeostasis.

### 3.3 Hormones and signaling pathway regulation

The gut microbiota regulates bone homeostasis through multiple metabolite–hormone networks. In the tryptophan metabolic axis, microbiota-derived indole compounds activate the aryl hydrocarbon receptor (AhR), which enhances bone formation and inhibits bone resorption by restoring intestinal barrier integrity and stimulating the Wnt/β-catenin signaling pathway, promoting nuclear translocation of β-catenin [29]. In the serotonin biphasic axis, peripheral 5-HT from the gut inhibits osteoblast proliferation through Htr1b receptors and CREB signaling, whereas central 5-HT from the brain promotes bone formation via brain-derived neurotrophic factor (BDNF) signaling. Lrp5 regulates bone mass by suppressing Tph1 expression in the duodenum, thereby reducing peripheral 5-HT synthesis [39,66]. In terms of growth regulation, the microbiota indirectly regulates bone growth through the growth hormone (GH)/insulin-like growth factor 1 (IGF-1) cascade, in which IGF-1 plays a key role by activating the PI3K/Akt osteogenic pathway [67]. In bile acid metabolism, dysbiosis (↑*Roseburia*/↓*Streptococcus*) in PMOP patients is significantly associated with bile acid metabolic disorders, which affect the enterohepatic circulation of estrogen through the farnesoid X receptor (FXR), forming a “microbiota-bile acid-estrogen” vicious cycle [65,68,69]. Mechanistically, FXR promotes osteoblast differentiation by stabilizing RUNX2 through inhibiting Thoc6-mediated ubiquitination; the FXR agonist obeticholic acid (OCA) can improve bone loss in ovariectomized mice [70]. Activation of Takeda G protein-coupled receptor 5 (TGR5) inhibits osteoclastogenesis via the AMP-activated protein kinase (AMPK) signaling pathway; dual targeting of FXR/TGR5 is a promising therapeutic strategy for PMOP [71,72]. The microbiota-derived metabolite imidazole propionic acid (ImP) exacerbates the progression of osteoporosis by inhibiting the AMPK signaling pathway. At the bone cell level, AMPK activation promotes osteogenic differentiation through an autophagy-dependent pathway and inhibits RANKL-induced osteoclastogenesis by suppressing the nuclear translocation of NFATc1 [73]. These findings reveal the multi-dimensional integration of the hormone-microbiota network in bone metabolism.

### 3.4 Vagus nerve signaling mediation

**Fig. 2:**
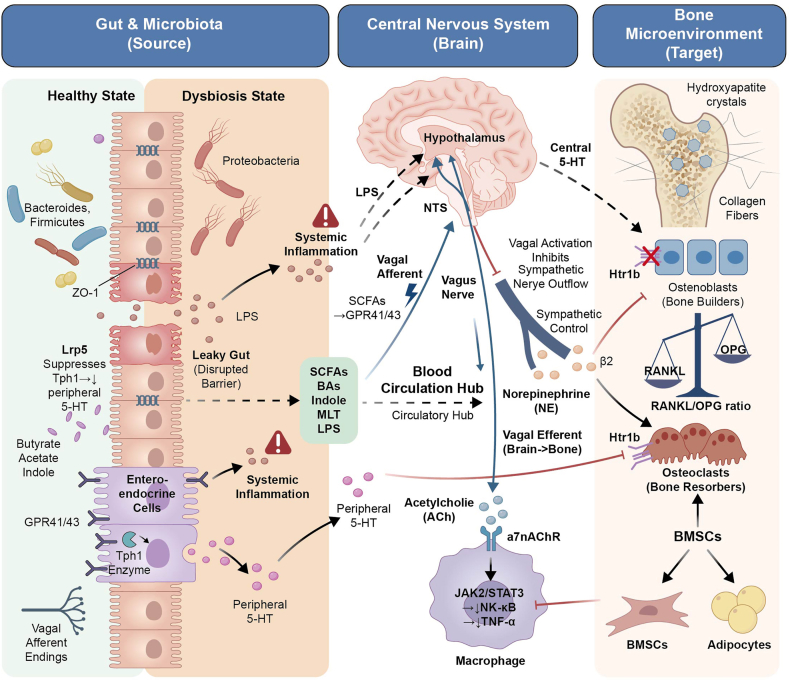
Gut microbiota orchestrates bone homeostasis through metabolic-immune-neural crosstalk.

The vagus nerve serves as the core neural pathway of the “gut-brain-bone axis.” The signaling cascade is divided into afferent and efferent pathways (Fig. 2).

**Afferent pathway:** Short-chain fatty acids (SCFAs), particularly butyrate, activate GPR41/43 on enteroendocrine cells and vagal afferent terminals [74]. These Gαi/o-coupled receptors inhibit adenylyl cyclase, reducing intracellular cAMP. The decrease in cAMP closes potassium channels, causing membrane depolarization, which opens voltage-gated calcium channels and allows Ca^2+^ influx. Elevated intracellular Ca^2+^ increases vagal afferent firing and triggers ERK1/2 phosphorylation in the nodose ganglia and nucleus tractus solitarius (NTS). The NTS relays signals to the hypothalamus [54,75].

**Efferent pathway:** Following central integration, efferent vagal fibers release acetylcholine, which binds to α7 nicotinic acetylcholine receptors (α7nAChR) on macrophages. α7nAChR activation triggers the JAK2/STAT3 pathway [76], suppressing nuclear translocation of NF-κB and reducing TNF-α production. Lower TNF-α increases the OPG/RANKL ratio in osteoblasts, shifting the balance toward reduced osteoclast formation [[77], [78], [79]].

**Sympathetic integration:** Vagal activation also reduces sympathetic outflow [80]. Sympathetic norepinephrine binds to β_2_-adrenergic receptors on osteoblasts, suppressing bone formation and promoting RANKL expression [81]. By dampening sympathetic tone, vagal signaling relieves this inhibition and limits RANKL-driven osteoclastogenesis [82]. This dual mechanism supports non-invasive neuromodulatory strategies (e.g., transcutaneous vagus nerve stimulation (tVNS)) for osteoporosis [83]. (see Table 2).

**Table 1:** Characteristics of gut microbiota dysbiosis associated with skeletal disorders.

Disease type	Microbial dysbiosis features	Core pathological mechanisms	Interventions and efficacy	Evidence level
OP	• ↑*Bacteroidetes*/*Firmicutes* ratio • ↓*Roseburia* • ↑*Klebsiella* • ↓*Akkermansia*	• **Intestinal barrier disruption** ↓ZO-1/Occludin → endotoxin translocation → TNF-α/IL-17↑ → RANKL/NF-κB activation [84] • **Reduced SCFAs synthesis** Treg suppression → downregulation of bone formation signaling [15,16,31,65]	• ***Akkermansia* transplantation:** Increased BMD (enhances barrier + SCFAs↑) [85]	Human cohort [86]; Animal model [87]
Impaired Fracture Healing	• ↓Microbial diversity • ↑F/B ratio • ↓*Lachnospiraceae*	• LPS translocation → TLR4/MyD88 activation → TNF-α/IL-6↑ → suppressed osteogenic differentiation + promoted bone marrow adiposity [61,62,88]	• **FOS/GOS:** Restored bone density (remodels microbiota + butyrate↑ + inhibits inflammation) [62]	Animal model [62]
RA	• ↑*Prevotella copri* • ↓*Faecalibacterium prausnitzii*	• SCFAs↓ → Treg/Th17 dysbiosis → IL-17↑ → Synovial inflammation • Intestinal barrier damage → bacterial metabolite translocation → autoimmune activation [40,89]	• **Probiotics** (*Lactobacillus + Bifidobacterium*): Reduced joint swelling [90] • **Butyrate:** Increased Treg cells (inhibits Th17) [91]	Human RCT [90]
GAON	• ↓*Lachnospiraceae*	• Reduced *Lachnospiraceae* → decreased butyrate → increased inflammatory cytokines (TNF-α/IL-6) → osteoclast activation → increased osteonecrosis risk [92,93]	• **Butyrate supplementation:** Bone protective effects (modulates microbiota + inhibits inflammation + promotes bone formation) [38,94,95]	Human cohort + Animal model [96]

**Footnotes:** BMD, bone mineral density; F/B, Firmicutes/Bacteroidetes ratio; FOS/GOS, fructooligosaccharides/galactooligosaccharides; GAON, glucocorticoid-associated osteonecrosis; HFD, high-fat diet; IL-6, interleukin-6; IL-17, interleukin-17; LPS, lipopolysaccharide; MyD88, myeloid differentiation primary response 88; NF-κB, nuclear factor κB; OP, osteoporosis; OVX, ovariectomized; PMOP, postmenopausal osteoporosis; RA, rheumatoid arthritis; RANKL, receptor activator of NF-κB ligand; RCT, randomized controlled trial; SCFAs, short-chain fatty acids; TLR4, Toll-like receptor 4; TNF-α, tumor necrosis factor α; Treg, regulatory T cell.

**Table 2:** Gut microbiota-targeted intervention strategies for bone metabolism.

Intervention Approach	Representative Agents/Methods	Mechanisms of Action	Bone Metabolism Improvement Indicators	Research Stage	Limitations
Probiotics	*Bifidobacterium lactis* Probio-M8	• Increases SCFAs → activates GPR41/43 to inhibit osteoclastogenesis [97] • Restores intestinal barrier → reduces LPS translocation [97,98]	BMD↑ Trabecular number↑ [99]	Phase II clinical trial	Strong strain specificity Poor colonisation stability
Prebiotics	FOS/GOS	• Enriches *Lachnospiraceae* → butyrate↑ • Inhibits HDAC → promotes osteogenic gene expression [62,100]	Bone loss↓ Serum RANKL↓ [62,101]	Phase I/II clinical trial	High inter-individual response variability
Synbiotics	Probiotics + Prebiotics	• Synergistically increases SCFAs-producing bacteria • ω-3 fatty acids → inhibits NF-κB inflammatory pathway [102,103]	PINP (bone formation marker)↑ [104]	Observational study	Limited regional dietary adaptability
FMT	Healthy donor microbiota (enriched with *Akkermansia*)	• Reshapes SCFAs/GPR41/IGF-1 axis • Promotes osteogenic differentiation [105,106]	Bone loss (OVX mice)↓ [107,108,109]	Animal models	Long-term safety unknown
Engineered preparations	Propolis nanoemulsions (PNEs)	• Enhances intestinal retention • Modulates *Streptococcus* abundance • Activates mTOR osteogenic pathway [110]	Osteogenic activity↑ [111,112]	Preclinical	High cost of scaled-up production
Dietary interventions	High-fiber diet	• SCFAs↑ • Inhibits osteoclast activity [113,114,115]	Bone mineralization density↑ [116,117]	Population cohort	Difficult to control adherence

**Footnotes:** BMD, bone mineral density; FMT, fecal microbiota transplantation; FOS/GOS, fructooligosaccharides/galactooligosaccharides; GPR41/43, G protein-coupled receptors 41/43; HDAC, histone deacetylase; IGF-1, insulin-like growth factor 1; LPS, lipopolysaccharide; mTOR, mammalian target of rapamycin; NF-κB, nuclear factor κB; OVX, ovariectomized; PINP, procollagen type I N-terminal propeptide; PNEs, propolis nanoemulsions; RANKL, receptor activator of NF-κB ligand; SCFAs, short-chain fatty acids.

## 4 The relationship between gut microbiota dysbiosis and bone diseases (Table 1)

### 4.1 OP

As elaborated in Section 3, gut microbiota dysbiosis critically drives OP progression, characterized by depletion of protective genera (e.g., *Akkermansia*) and enrichment of pathobionts (*Klebsiella, Escherichia-Shigella*), potentially serving as 16 S rRNA-based diagnostic biomarkers [31,85]. In postmenopausal OP (PMOP), dysbiosis (↓diversity, a*ltered Roseburia/Streptococcus ratio*) triggers a triple pathological cascade: ① gut barrier disruption (↓ZO-1/Occludin) enabling endotoxin translocation and systemic inflammation [65]; ② disrupted tryptophan metabolism impairs intestinal barrier repair and metabolic regulation [1,10,[118], [119], [120]]; and ③ immune dysbiosis where pro-inflammatory cytokines (TNF-α, IL-17) activate RANKL signaling to accelerate bone resorption [15,121,122]. Targeted interventions confirm that MLT supplementation or probiotics restore microbial ecology and elevate SCFAs, significantly improving bone microarchitecture [[97], [104], [123], [124]]). Future studies must leverage multi-omics technologies to elucidate strain-specific functions and advance microbial biomarker translation into clinical practice [125]. Thus, OP-associated dysbiosis involves concurrent loss of protective taxa and expansion of pathobionts, driving bone loss via three synergistic pathways, gut barrier disruption, tryptophan metabolic dysregulation, and immune activation, suggesting that multi-node rather than single-microbe targeting is required.

### 4.2 Other arthritic and spine-related disorders

A dysbiosis in gut microbiota is strongly associated with the development and advancement of rheumatoid arthritis (RA) [126,127], characterized by reduced microbial diversity, aberrant enrichment of the pathobiont *Prevotella copri* in early RA patients, and significant depletion of the beneficial bacterium *Faecalibacterium prausnitzii* [[89], [128], [129], [130]]. This dysbiosis exacerbates disease through a dual pathological mechanism: first, diminished SCFAs synthesis impairs butyrate-mediated immunomodulatory functions, specifically reducing the ability to suppress Treg differentiation and promote Th17 polarization, thereby disrupting immune homeostasis [40,91]; second, impaired intestinal barrier function facilitates translocation of bacterial metabolites, activating immune cells like macrophages to release pro-inflammatory cytokines such as TNF-α and IL-1β, triggering a systemic inflammatory cascade [131,132]. Intervention studies confirm that probiotics, prebiotics, and pharmacologic agents can partially restore gut microbiota balance in RA patients, ameliorating disease symptoms [90,133]. Collectively, these results emphasize the essential function of gut microbiota through the “gut-bone axis” in various bone-related disorders, providing a crucial foundation for novel therapeutic strategies.

Beyond RA, gut microbiota dysbiosis has been increasingly implicated in other arthritic and spine-related disorders. In osteoarthritis (OA), altered gut microbial composition (e.g., reduced Akkermansia and increased pro-inflammatory taxa) is associated with synovial inflammation and cartilage degradation [41,134]. For psoriatic arthritis (PsA), metagenomic studies have identified a distinct gut microbial signature (e.g., Bacteroides network) correlated with disease activity [135]. In gouty arthritis, gut dysbiosis contributes to hyperuricemia by modulating purine metabolism and urate excretion [136]. Regarding spine-related disorders, a systematic review and meta-analysis of 47 studies confirmed significant gut microbial diversity reduction in ankylosing spondylitis (AS), with decreased Bifidobacterium and increased Bacteroidetes abundance [137]. For intervertebral disc degeneration (IDD), preclinical evidence suggests that gut dysbiosis-induced systemic inflammation accelerates disc matrix breakdown via immunomodulation and bacterial translocation [138]. Finally, large cohort studies have demonstrated that lower gut microbial diversity is associated with increased risk of incident fractures [139], and specific SCFA signatures correlate with lumbar spine bone mineral density (BMD) in postmenopausal women [140], supporting a role for gut microbiota in spinal osteoporotic fracture risk. These findings extend the gut-bone axis concept to a broader spectrum of musculoskeletal disorders and highlight disease-specific microbiota signatures as potential therapeutic targets.

## 5 Advances in animal and clinical research

### 5.1 Evidence from animal models

Studies in animal models provide critical evidence for the gut microbiota-bone metabolism connection. Research using murine models demonstrates significant alterations in gut microbiota structure during osteoporosis, characterized by a markedly increased F/B ratio. Analysis of fecal and serum metabolites via ultra-high performance liquid chromatography–mass spectrometry (UHPLC-MS) further reveals substantial differences in bile acid metabolism between osteoporotic and control groups [141]. Similarly, a study focusing on disuse osteoporosis in rat models observes significant changes in microbial abundance and fecal metabolic profiles, identifying aberrant activation of bile acid metabolism pathways and shifts in bone mineral density (BMD)-associated bacterial taxa [142]. Collectively, evidence from these diverse animal models substantiates the pivotal role of the “gut-bone axis” in bone metabolism regulation, establishing a robust experimental foundation for microbiota-targeted interventions.

### 5.2 Current status and limitations of clinical research

Further clinical research supports the link between gut microbiota and osteoporosis but faces substantial limitations that require critical appraisal. First, heterogeneity in study design severely constrains data interpretation. The majority of existing evidence derives from cross-sectional studies [86], which can identify associations but cannot establish temporal or causal relationships between dysbiosis and bone loss. Longitudinal cohort studies are scarce, and randomized controlled trials (RCTs) targeting gut microbiota modulation for bone outcomes remain in early phases (mostly Phase I/II) with small sample sizes and short follow-up [143,144]. The lack of large-scale, prospective RCTs precludes definitive conclusions about efficacy and safety of microbiota-based interventions.

Second, inadequate control for confounding factors introduces substantial bias. Critical confounders such as dietary intake (fiber, calcium, vitamin D), age, menopausal status, medications (bisphosphonates, denosumab, proton pump inhibitors, antibiotics), and comorbidities (diabetes, obesity, inflammatory bowel disease) are often poorly documented or unevenly distributed across study groups [145,98]. For example, dietary fiber intake directly shapes SCFA production and gut microbiota composition, yet many studies fail to collect or adjust for nutritional data. Similarly, the use of proton pump inhibitors is known to alter gut microbial communities and may independently affect bone mineral density, but such medication histories are frequently omitted from analyses.

Third, methodological variability in microbiome sequencing and analysis hampers cross-study comparisons. Studies employ different protocols for 16 S rRNA gene sequencing (variable regions, primers, sequencing platforms), bioinformatics pipelines (QIIME, DADA2, UPARSE), and reference databases (Greengenes, SILVA, RDP). These technical differences lead to inconsistent taxonomic resolution and batch effects that can obscure true biological signals [146]. Furthermore, the lack of standardized sample collection, storage, and DNA extraction methods across centers adds additional variability. Metagenomic sequencing, which offers functional insights, remains underutilized in most clinical bone studies [147].

Fourth, causal inference is severely limited by current designs. Cross-sectional studies cannot differentiate whether dysbiosis is a cause or consequence of osteoporosis. While animal models demonstrate causal roles for specific microbes via FMT or gnotobiotic approaches [87], human data are almost exclusively correlative. Reverse causality—where osteoporosis or its treatments alter gut microbiota rather than the reverse—cannot be excluded. Mendelian randomization (MR) studies have recently provided genetic evidence supporting a causal link between certain gut microbial taxa and BMD [87], but such approaches are few and require validation in independent cohorts.

Additionally, contradictory findings exist. For instance, acetate promotes adipogenesis over osteogenesis via GPR41/43 [52], and some trials report no significant BMD improvement with probiotics. Acknowledging such negative evidence is essential for a balanced view of the gut-bone axis.

To address these limitations and enable personalized bone health strategies, future investigations should adopt prospective longitudinal designs with repeated sampling, rigorously control for confounders (diet, medications, comorbidities), standardize microbiome protocols (including negative controls and spike-ins), and prioritize large-scale RCTs with bone-specific endpoints. Furthermore, integrating multi-omics technologies (metagenomics, metabolomics) and Mendelian randomization will strengthen causal inference. This will advance the translation of personalized microbiota-based treatments into clinical settings.

## 6 Potential strategies for modulating gut microbiota to improve bone metabolism

### 6.1 Application of probiotics and prebiotics

Multiple studies confirm that probiotics regulate bone metabolism through multiple pathways [54,[148], [149], [150]]. The detailed mechanisms—including SCFA production, intestinal barrier strengthening, and immune modulation—are systematically discussed in Section 3. For instance, specific probiotics elevate SCFAs levels, which demonstrably influence BMD by balancing bone resorption and formation [2,45,120,100,151]. Conversely, prebiotics—non-digestible carbohydrates—selectively stimulate beneficial gut bacteria proliferation, indirectly modulating bone metabolism. Research indicates prebiotics improve BMD and bone strength by altering microbial structure/function to enhance calcium absorption/utilization [104,[102], [152], [153], [154]]. Furthermore, synbiotics (probiotic-prebiotic combinations) exhibit synergistic effects in bone health, more effectively optimizing microbial homeostasis, reinforcing the intestinal barrier, and improving bone metabolism via gut-bone axis signaling [148,152,155,113]. Probiotic efficacy depends on baseline microbiota and metabolism.

### 6.2 Dietary interventions

Dietary interventions influence bone metabolism by altering the composition and metabolic functions of gut microbiota. High-fiber diets and fermented foods favorably influence bone metabolism through distinct mechanisms. High-fiber diets significantly increase microbial diversity and abundance, promoting beneficial bacteria (e.g., Bifidobacterium and Lactobacillus) and suppressing potential pathobionts, thereby improving gut microecology [156,157]. These bacteria break down dietary fiber to generate SCFAs, such as butyrate, which help preserve the intestinal barrier and enhance bone health by influencing immune reactions and reducing inflammation [158,159] Fermented foods deliver active probiotics that colonize the gut, interact with resident microbiota, and selectively stimulate beneficial bacteria while inhibiting harmful species [160,161]. Organic acids (e.g., lactate) generated during fermentation further optimize the gut environment to indirectly regulate bone metabolism [116]. These effects operate within the gut-bone axis framework—microbiota alterations impact calcium absorption efficiency and skeletal mineralization processes, ultimately modulating BMD and bone strength [104,105]. Consequently, microbiota-targeted dietary strategies benefit not only intestinal health but may also prevent skeletal disorders like osteoporosis through bone metabolic pathway modulation.

### 6.3 Exploration of fecal microbiota transplantation (FMT)

FMT, an emerging therapeutic strategy, demonstrates potential for bone metabolism regulation. Research indicates that FMT ameliorates bone loss by remodeling gut microbiota structure and function. The underlying mechanisms include enhanced intestinal barrier integrity and elevated SCFA levels, which confer osteoprotective effects [87,162,106]. For instance, in ovariectomized (OVX) -induced osteoporotic mouse models, FMT effectively suppressed osteoclast overactivation and significantly attenuated bone loss [87,107]. Furthermore, FMT shows promise for bone metabolism-related disorders: studies in sickle cell disease mouse models reveal FMT improves bone loss via modulation of the SCFA/GPR41/IGF-1 signaling axis, suggesting healthy microbiota may protect bone through IGF-1 regulation [106]. In aged rat models, FMT mitigated bone loss by optimizing microbial diversity and composition—effects closely associated with improved gut barrier function, further evidencing its multi-pathway regulatory potential [162]. Notably, FMT may indirectly influence bone homeostasis through pathways like bile acid metabolism modulation [163]. Collectively, although current evidence primarily derives from animal models, FMT exhibits significant therapeutic potential for bone metabolism, providing a crucial scientific foundation for future clinical interventions in bone-related disorders [164,165]. Subsequent research should focus on elucidating long-term efficacy, safety, and precise mechanisms in diverse human populations.

However, translation of FMT and probiotics for bone diseases faces practical barriers: standardized donor selection criteria for FMT remain lacking [166]; optimal dosing regimens (frequency, dosage, route) are undefined [167]; and long-term safety data (e.g., antibiotic resistance, dysbiosis rebound) are absent [168]. Similar issues exist for probiotics regarding strain specificity and formulation standardization. Overcoming these requires bone-specific trials and harmonized protocols.

## 7 Conclusions and perspectives

Research on the gut-bone axis has significantly expanded our understanding of skeletal disorders like osteoporosis, highlighting the promise of microbiome-targeted interventions for prevention and therapy. However, translating fundamental insights into clinical applications faces substantial hurdles. A primary challenge is the heterogeneity of existing studies. Although evidence implicates specific microbes (e.g., *Lactobacillus, Bifidobacterium*) and SCFAs in BMD regulation [120,104,169,170], divergent experimental models, limited sample sizes, and significant inter-individual microbiota variation often lead to inconsistent findings [171]. This necessitates standardized methodologies and the integration of multi-omics approaches (metagenomics, metabolomics) [[172], [173], [174], [175], [176]] to precisely map dynamic host-microbiota interactions. A second critical bottleneck is the incomplete elucidation of molecular mechanisms. Although hypotheses, such as SCFAs (e.g., butyrate) modulating bone resorption via Treg/Th17 balance, have preliminary support [22], downstream signaling pathways require clarification using tools like tissue-specific knockout models. Moreover, causal networks linking the microbiota to key bone-regulating pathways remain largely undefined. These include bile acid modification of estrogen enterohepatic cycling and microbiota-dependent regulation of vitamin D active metabolites. Emerging areas, such as vagus nerve-mediated gut-brain-bone signaling and microbiota-metabolite modulation of bone sympathetic tone [29,177,178], demand dedicated investigation. Critically, the lack of strong human data hinders clinical translation. Research on animals shows that probiotics and FMT are effective in reducing bone loss, yet human research remains largely observational or confined to small-scale trials [54], lacking large-scale randomized controlled trials (RCTs) to confirm long-term effectiveness and safety. The significant differences in microbiota composition among individuals result in unpredictable treatment outcomes, and the lack of standardized procedures for FMT donor screening, probiotic strain selection/formulation, and SCFA detection adds to the challenges. Overcoming these barriers requires stepwise clinical trials and the development of host-microbiota phenotyping systems to enable personalized approaches [179].

To provide a more actionable roadmap, we propose specific testable priorities: (1) For osteoporosis risk stratification, the most promising microbial biomarkers ready for validation include *Akkermansia muciniphila* abundance, the Roseburia/*Streptococcus ratio*, and serum butyrate levels. (2) For the first large-scale FMT RCT in postmenopausal osteoporosis, primary endpoints should include percentage change in lumbar spine BMD at 12 months, incidence of fragility fractures at 24 months, and safety outcomes. Secondary endpoints should include changes in gut microbiota diversity, SCFA profiles, and bone turnover markers (procollagen type I N-terminal propeptide (PINP), C-terminal telopeptide of type I collagen (CTX-1)).

Future research must prioritize multi-dimensional innovation to bridge these gaps. Mechanistically, advanced tools such as spatiotemporal omics and organoid co-culture systems are crucial to dissect direct interactions between microbial metabolites and bone cells [180]. Translationally, developing microbiome-metabolite diagnostic panels for osteoporosis risk stratification and engineering bacterial systems for targeted delivery of osteoprotective metabolites represent essential goals. Concurrently, integrating validated microbiome interventions into comprehensive osteoporosis management guidelines and establishing robust cross-disciplinary frameworks—encompassing microbiology, bone biology, immunology, and neuroendocrinology—is paramount for advancing the field.

Collectively, realizing the therapeutic potential of the gut-bone axis requires balancing deep mechanistic inquiry with efficient clinical translation. Only through concerted efforts integrating multi-omics technologies, rigorously designed clinical trials, and synergistic cross-disciplinary collaboration can the field progress from foundational “microbiome theory” towards the realization of “precision skeletal medicine," ultimately delivering transformative strategies for osteoporosis and related disorders.

## Declaration of generative AI in scientific writing

During the preparation of this work, the authors used AI to assist with language refinement and polishing. After using this tool, the authors reviewed and edited the content as needed and take full responsibility for the content of the published article.

## Funding

The work completed with the support from the Yunnan Fundamental Research Kunming Medical University Projects (202001AY070001-266), Kunming Health Science and Technology Personnel Training Project-Medical science and technology leader (2023-SW (Lead)-15), Yunnan Provincial Education Department Scientific Research Fund (2024J0285), Science and Technology Talent and Platform Program of Yunnan Provincial Science and Technology Department (Technological Innovation Talent) (202405AD350091), Yunnan Science and Technology Talent and Platform Program (Academician Expert Workstation) (202405AF140037), Yunnan Fundamental Research Kunming Medical University Projects (202301AY070001-288), Yunnan Fundamental Research Kunming Medical University Projects (202301AY070001-268), Kunming Municipal Reserve Talents Program for Medical Science Disciplines (2024-SW (Reserve)-57), Kunming Municipal Health Commission Health Research Grant Project (2023-04-07-010).

## CRediT authorship contribution statement

**Rong Qin:** Conceptualization, Funding acquisition, Writing – review & editing. **Peng Yu:** Visualization, Writing – review & editing. **Hui Wang:** Writing – original draft, Methodology. **Jing Zhou:** Resources, Writing – review & editing. **Rui Gong:** Writing – review & editing, Validation. **Yiyao Duan:** Resources, Writing – review & editing. **Hongping Jia:** Writing – original draft, Conceptualization. **Mingzhu Xie:** Resources, Writing – review & editing. **Yucheng Zhou:** Supervision, Writing – review & editing. **Jun Hu:** Conceptualization, Writing – review & editing.

## Declaration of competing interest

The authors declare that they have no known competing financial interests or personal relationships that could have appeared to influence the work reported in this paper.

## Abbreviations

α7nAChR: alpha7 nicotinic acetylcholine receptor
AhR: Aryl hydrocarbon receptor
BAs: bile acids
AMPK: AMP-activated protein kinase
BDNF: brain-derived neurotrophic factor
BMD: Bone mineral density
CTX-1: C-terminal telopeptide of type I collagen
F/B: Firmicutes/Bacteroidetes ratio
FMT: Fecal microbiota transplantation
FOS/GOS: Fructooligosaccharides/Galactooligosaccharides
FXR: farnesoid X receptor
GAON: Glucocorticoid-associated osteonecrosis
GH: growth hormone
GPR43/41: G protein-coupled receptors 43/41
HDACs: Histone deacetylases
HFD: High-fat diet
IL-6: interleukin-6
IL-17: interleukin-17
IGF-1: insulin-like growth factor 1
ImP: imidazole propionic acid
LPS: Lipopolysaccharide
MLT: Melatonin
MSCs: mesenchymal stem cells
mTOR: mammalian target of rapamycin
MyD88: Myeloid differentiation primary response 88
MR: Mendelian randomization
NFATc1: nuclear factor of activated T cells c1
NF-κB: Nuclear factor κB
NTS: nucleus tractus solitarius
OCA: obeticholic acid
OP: Osteoporosis
OPG: Osteoprotegerin
OVX: Ovariectomized
PINP: procollagen type I N-terminal propeptide
PMOP: Postmenopausal osteoporosis
PNEs: propolis nanoemulsions
RA: Rheumatoid arthritis
RANKL: Receptor activator of NF-κB ligand
RCT: Randomized controlled trial
SCFAs: Short-chain fatty acids
Th17: T helper 17 cells
TLR4: Toll-like receptor 4
TGR5: Takeda G protein-coupled receptor 5
TNF-α: tumor necrosis factor α
tVNS: Transcutaneous vagus nerve stimulation
Treg: regulatory T cells
UHPLC-MS: Ultra-high performance liquid chromatography-mass spectrometry
UV: Ultraviolet.
